# Do the Effects of Resveratrol on Thermogenic and Oxidative Capacities in IBAT and Skeletal Muscle Depend on Feeding Conditions?

**DOI:** 10.3390/nu10101446

**Published:** 2018-10-06

**Authors:** Iñaki Milton-Laskibar, Leixuri Aguirre, Usune Etxeberria, Fermin I. Milagro, J. Alfredo Martínez, Maria P. Portillo

**Affiliations:** 1 Nutrition and Obesity Group, Department of Nutrition and Food Science, Lucio Lascaray Research Institute, University of the Basque Country (UPV/EHU), 01006 Vitoria, Spain; inaki.milton@ehu.eus (I.M.-L.); mariapuy.portillo@ehu.eus (M.P.P.); 2 CIBERobn Physiopathology of Obesity and Nutrition, Institute of Health Carlos III, 28029 Madrid, Spain; fmilagro@unav.es (F.I.M.); jalfmtz@unav.es (J.A.M.); 3 BCC Innovation, Technological Center of Gastronomy, 20009 Donostia, Spain; uetxeberria@bculinary.com; 4 Basque Culinary Center, Mondragon Unibertsitatea, 20009 San Sebastián, Spain; 5 Department of Nutrition, Food Sciences and Physiology, Centre for Nutrition Research, University of Navarra, 31008 Pamplona, Spain; 6 IMDEA Food, 28049 Madrid, Spain

**Keywords:** resveratrol, energy restriction, thermogenesis, high-fat high-sucrose diet, rat

## Abstract

The aim of this study was to compare the effects of mild energy restriction and resveratrol on thermogenic and oxidative capacity in interscapular brown adipose tissue (IBAT) and in skeletal muscle. Rats were fed a high-fat high-sucrose diet for six weeks, and divided into four experimental groups fed a standard diet: a control group, a resveratrol-treated group, an energy-restricted group and an energy-restricted group treated with resveratrol. Weights of IBAT, gastrocnemius muscle and fat depots were measured. Activities of carnitine palmitoyltransferase (CPT) and citrate synthase (CS), protein levels of sirtuin (SIRT1 and 3), uncoupling proteins (UCP1 and 3), glucose transporter (GLUT4), mitochondrial transcription factor (TFAM), nuclear respiratory factor (NRF1), peroxisome proliferator-activated receptor (PPARα) and AMP activated protein kinase (AMPK) and peroxisome proliferator-activated receptor gamma coactivator (PGC1α) activation were measured. No changes in IBAT and gastrocnemius weights were found. Energy-restriction, but not resveratrol, decreased the weights of adipose depots. In IBAT, resveratrol enhanced thermogenesis activating the SIRT1/PGC1α/PPARα axis. Resveratrol also induced fatty acid oxidation and glucose uptake. These effects were similar when resveratrol was combined with energy restriction. In the case of gastrocnemius muscle, the effects were not as clear as in the case of IBAT. In this tissue, resveratrol increased oxidative capacity. The combination of resveratrol and energy restriction seemingly did not improve the effects induced by the polyphenol alone.

## 1. Introduction

The phenomenon of adaptive thermogenesis, also known as non-shivering or facultative thermogenesis, is a phenomenon characterized by heat production in response to environmental temperature or diet [1]. It is mediated by the action of uncoupling proteins (UCPs), a type of protein embedded in the inner mitochondrial membrane which uncouples the oxidative phosphorylation. As a result, the proton gradient is dissipated and used to produce heat instead of synthetizing adenosine triphosphate (ATP) [2]. In this regard, brown adipose tissue (BAT) has been identified as the main thermogenic tissue, due to its high content in mitochondria [3,4]. This tissue was thought to exist only in small mammals and newborns, but the recent discovery of BAT in adult humans has reactivated the interest of the scientific community in this tissue as a possible means of body weight management [5,6,7].

To produce heat, the lipids stored in BAT are firstly used as substrates [8] and then, in order to maintain this thermogenic activity, triglycerides from circulation are used [9]. Moreover, circulating glucose is also used by BAT as a substrate for thermogenesis [9]. As a result, both plasma triglyceride levels as well as plasma glucose levels are decreased by BAT thermogenic activity [9]. All these observations highlighted BAT as an interesting target-tissue for obesity and metabolic syndrome management.

Along with BAT, the other main thermogenic tissue in human body is skeletal muscle, which is the largest organ of the human body and determines the basal metabolic rate [10,11]. UCP3 is the characteristic uncoupling protein present in this tissue [12]. Although UCP3 was at first thought to participate in thermogenesis and energy expenditure (as does its more abundant ortholog UCP1, present in BAT), it has been proposed that its function is more related to glucose and fatty acid metabolism [13,14]. 

As previously mentioned, diet is one of the main modulators of thermogenesis, mediating this process in two different ways. It has been reported that macronutrient composition of the diet can influence thermogenic activity in animal models not only in BAT, but also in skeletal muscle [15]. Moreover, a reduction in energy intake has also been related to thermogenesis regulation. In this regard, while excess food intake (hyperphagia) has been related to increased thermogenesis (diet-induced thermogenesis), reductions in UCP1 mRNA in BAT have been identified in conditions of food scarcity [16]. 

In recent years interest in natural compounds with positive health effects has increased within scientific community. One of these bioactive compounds, resveratrol (3,5,4′-trihydroxy-*trans*-stilbene), a phenolic compound belonging to the stilbene group, has attracted a great deal of attention [17,18,19]. As reported by several authors, resveratrol is able to mimic the effects induced by dietary energy restriction without reducing caloric intake [20,21]. In addition, many studies have described resveratrol as a potential inducer of thermogenesis in rodent models fed obesogenic [22] or standard [23] diets. 

In this context, the present study aimed to compare the effects of mild energy restriction and resveratrol on thermogenic capacity of BAT and skeletal muscle, in terms of efficiency and mechanisms of action. Moreover, the capacity of resveratrol to induce thermogenesis under energy restriction feeding conditions was also studied.

## 2. Material and Methods

### 2.1. Animals, Diets and Experimental Design

The experiment was conducted using 36 six-week-old male Wistar rats (Harlan Ibérica, Barcelona, Spain), and conducted in accordance with the University of the Basque Country’s Guide for the Care and Use of Laboratory Animals (Reference protocol approval M20_2016_039). The rats, individually housed in polycarbonate metabolic cages (Tecniplast Gazzada, Buguggiate, Italy), were placed in a controlled air-conditioned room (22 ± 2 °C) with a 12-h light-dark cycle. After a 6-day adaptation period, animals were fed a high-fat high-sucrose (HFHS) diet (OpenSource Diets, Lynge, Denmark; Ref. D12451) for 6 weeks (45% of energy as fat, 13% of energy as sucrose and 4.7 kcal/g of energy) (Table S1 A). After this period, the animals were fed over 6 additional weeks with a standard semi-purified diet (OpenSource Diets, Lynge, Denmark; Ref. D10012G) which provided 3.9 kcal/g, 16% as fat, 64% as carbohydrates and 20% as protein (Table S1 B), and randomly distributed into four experimental groups (*n* = 9): the control group (C), the resveratrol group (RSV), the restricted group (R) and the combined group (RR) (Figure S1). The RSV group was treated with a dose of 30 mg/kg body weight/day of resveratrol, the R group was submitted to a mild energy restriction of 15% of total daily energy intake and the combined group was submitted to a mild energy restriction (15%) and treated with resveratrol (30 mg/kg body weight/day). In the case of C and RSV groups, the animals had free access to food and water (ad libitum). The selected resveratrol dose was based on the previous experience of our group [24]. In order to calculate the exact diet amount provided to the animals in the restricted groups, the spontaneous food intake of the C group rats was taken into account. 

Once the whole experimental period (12 weeks) was completed, animals from the four experimental groups were sacrificed after an overnight fasting (12 h) under anesthesia (chloral hydrate) by cardiac exsanguination. Interscapular BAT (IBAT), gastrocnemius muscles and white adipose tissue depots from different anatomical locations (subcutaneous, mesenteric, perirenal and epididymal) were dissected, weighed, and immediately frozen in liquid nitrogen. All the samples were stored at −80 °C until analysis.

### 2.2. Enzyme Activities

The activity of carnitine palmitoyltransferase-1a and b (CPT-1a and CPT-1b) was measured spectrophotometrically in the mitochondrial fraction of IBAT and gastrocnemius muscle, respectively. Briefly, tissue samples (200 mg) were homogenized in 3 volumes (*wt*/*vol*) of buffer containing 10 mmol/L Tris-HCl, 1 mmol/L EDTA and 0.25 mol/L sucrose (pH 7.4), and then centrifuged (700× *g* for 10 min at 4 °C). After this first centrifugation, the supernatants were collected and centrifuged again (12,000× *g* for 15 min at 4 °C). The pellets were resuspended in resuspension buffer (220 mmol/L mannitol, 70 mmol/L sucrose, 1 mmol/L ethylenediaminetetraacetic acid (EDTA) and 2 mmol/L 4-(2-Hydroxyethyl)piperazine-1-ethanesulfonic acid (HEPES), pH 7.4), and the protein content determined according to the Bradford method [25]. The activity of CPT-1a and CPT-1b was measured by using the Bieber et al. method [26,27]. The activity of the enzyme was represented as nanomoles CoA formed per minute, per milligram of protein.

In the case of citrate synthase (CS), activity was also assessed spectrophotometrically, following the Srere method [27] by measuring the appearance of free CoA. Briefly, frozen IBAT and gastrocnemius muscle samples (50 and 100 mg respectively) were homogenized in 25 vol (*wt*/*vol*) of 0.1 M Tris-HCl buffer (pH 8.0). Homogenates were incubated for 2 min at 30 °C with 0.1 M Tris-HCl buffer containing 0.1 mM 5,5′-dithio-bis-[2-nitrobenzoic acid] (DTNB), 0.25 Triton X-100, 0.5 mM oxalacetate and 0.31 mM acetyl CoA, and readings were taken at 412 nm. CS activity was expressed as nmol CoA formed per minute, per milligram of protein. The protein content of the samples was determined by the Bradford method [25], using bovine serum albumine as standard.

### 2.3. Western Blot

For sirtuin 1 and 3 (SIRT1 and SIRT3), AMP activated protein kinase (AMPK), glucose transporter 4 (GLUT4), mitochondrial transcription factor A (TFAM), uncoupling proteins 1 and 3 (UCP1 and UCP3) and Tubulin, IBAT and gastrocnemius muscle samples (100 mg) were homogenized in 1000 μL of cellular phosphate buffer saline (PBS) (pH 7.4), containing protease inhibitors (100 mM phenylmethylsulfonyl fluoride and 100 mM iodoacetamide). Homogenates were centrifuged at 800× *g* for 10 min at 4 °C. Protein concentration in homogenates was measured by Bradford method (Bradford et al., 1976) using bovine serum albumin as standard. In the case of peroxisome proliferator-activated receptor gamma coactivator 1-alpha (PGC1α), nuclear respiratory factor 1 (NRF1), peroxisome proliferator-activated receptor alpha and beta/delta (PPARα and PPARβ/δ) and histone H3 (Histone H3), nuclear protein extraction were carried out with 100 mg of IBAT and gastrocnemius muscle, as previously described [28]. 

Immunoblot analyses were carried out using 50 μg and 40 μg of IBAT and gastrocnemius muscle total protein extracts (respectively), and 35 μg and 60 μg of IBAT and gastrocnemius muscle nuclear protein extracts (respectively), separated by electrophoresis in precast 4–15% sodium dodecyl sulfate (SDS)-polyacrylamide gradient gels (Bio-Rad, Hercules, CA, USA) and transferred to PVDF membranes (Bio-Rad, Hercules, CA, USA). The membranes were then blocked with 5% caseine PBS-Tween buffer for 2 h at room temperature. Subsequently, they were blotted with the appropriate antibodies overnight at 4 °C. Protein levels were detected via specific antibodies (Table S2) for SIRT3 (1:500), UCP1 (1:500), UCP3 (1:500), TFAM (1:500), GLUT4 (1:5000), PPARβ/δ (1:500) (Santa Cruz Biotech, Dallas, TX, USA), AMPK (1:1000), Tubulin (1:5000), Histone H3 (1:1000) (Cell Signaling Technology, Danvers, MA, USA) and SIRT1 (1:1000), PGC1α (1:1000), NRF1 (1:1000), PPARα (1:1000) (Abcam, Cambridge, UK). Afterward, polyclonal anti-mouse for SIRT3 (1:5000) (Santa Cruz Biotech, Dallas, TX, USA), anti-goat for UCP1, UCP3, TFAM and GLUT4 (1:5000) (Santa Cruz Biotech, Dallas, TX, USA) and anti-rabbit for SIRT1, AMPK, Tubulin, Histone H3, PGC1α, NRF1, PPARα and PPAR β/δ (1:5000) (Santa Cruz Biotech, Dallas, TX, USA) were incubated for 2 h at room temperature, and the levels of the aforementioned proteins were measured (Table S2). After antibody stripping, the membranes were blocked and then incubated with phosphorylated AMPK (threonine 172, 1:1000), and acetylated lysine (1:1000) (Cell Signaling Technology, Danvers, MA, USA) antibodies (Table S2). The bound antibodies were visualized by an electrochemiluminescence (ECL) system (Thermo Fisher Scientific Inc., Rockford, IL, USA) and quantified by a ChemiDoc MP Imaging System (Bio-Rad, Hercules, CA, USA). Specific bands were identified by using a standard loading buffer (Precision Plus protein standards dual color; Ref. 161-0374 Bio-Rad). 

### 2.4. Statistical Analysis

Results are presented as mean ± standard error of the mean (SEM). Statistical analysis was performed using SPSS 21.0 (SPSS, Chicago, IL, USA). All the variables were normally-distributed according to the Shapiro-Wilks test. Data were analyzed by one-way analysis of variance (ANOVA) followed by the Newman–Keuls post hoc test. Significance was assessed at the *p* < 0.05 level.

## 3. Results

### 3.1. Body Weight, Food Intake, Adipose Tissue Weights, and Interscapular Brown Adipose Tissue (IBAT) and Skeletal Muscle Weights

At the end of the whole experimental period, as expected, significantly lower body weights were observed in the groups submitted to energy restriction (R and RR) compared with the C group with no differences among them. In the case of the RSV group, body weight was similar to that found in the C group [29]. In the same line, significantly lower food intakes were found in both restricted groups (R and RR) compared with the C group, without differences between them [30]. When the weights of the four white adipose depots were pooled, a similar pattern to that found in the body weights, lower values in the restricted groups (R and RR) and no changes in the group treated with resveratrol (RSV) were noted (Table 1). Finally, IBAT and gastrocnemious weights did not show significant differences among the four experimental groups (Table 1).

### 3.2. Enzyme Activities

**Figure 1:**
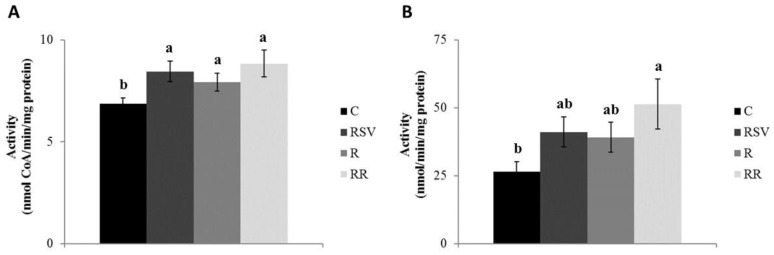
Activities of CPT-1a (**A**) and CS (**B**) in IBAT of rats fed an obesogenic diet for 6 weeks and then shifted to a standard diet (C), or standard diet supplemented with resveratrol (RSV), or submitted to energy restriction and fed a standard diet (R), or submitted to energy restriction and fed a standard diet supplemented with resveratrol (RR) (*n* = 9/group) for additional 6 weeks. Values are represented as means ± SEM. Differences among groups were determined by using one-way ANOVA followed by Newman–Keuls post hoc test. Values not sharing a common letter are significantly different (*p* < 0.05). CPT-1a: carnitine palmitoyltransferase-1a, CS: citrate synthase, IBAT: interscapular brown adipose tissue.

**Figure 2:**
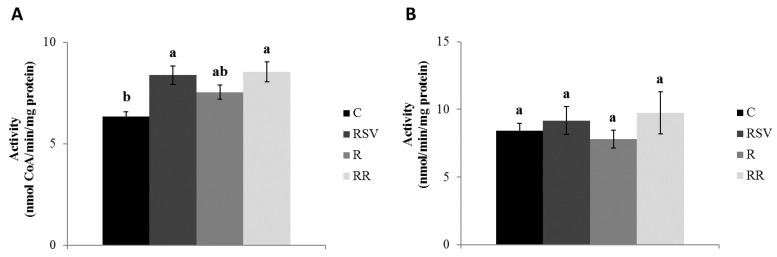
Activities of CPT-1b (**A**) and CS (**B**) in gastrocnemius muscle of rats fed an obesogenic diet for 6 weeks and then shifted to a standard diet (C), or standard diet supplemented with resveratrol (RSV), or submitted to energy restriction and fed a standard diet (R), or submitted to energy restriction and fed a standard diet supplemented with resveratrol (RR) (*n* = 9/group) for additional 6 weeks. Values are represented as means ± SEM. Differences among groups were determined by using one-way ANOVA followed by Newman–Keuls post hoc test. Values not sharing a common letter are significantly different (*p* < 0.05). CPT-1b: carnitine palmitoyltransferase-1b, CS: citrate synthase.

Regarding CPT-1a in IBAT, significantly greater enzyme activities were observed in the three treated groups when compared with the C group (*p* = 0.001 in RSV, *p* = 0.004 in R and *p* = 0.020 in RR), with no differences among them (Figure 1A). As far as gastrocnemius muscle is concerned, a statistically significant increase in the activity of CPT-1b enzyme was observed in the groups supplemented with resveratrol (RSV and RR) when compared with the C group (*p* = 0.017 and *p* = 0.023, respectively), while in the case of the R group a non significant trend towards a greater activity (+19.1%; *p* = 0.065) was observed (Figure 2A).

In the case of the CS, RSV and RR groups, these showed a significantly increased enzyme activity in IBAT when compared with the C group (*p* = 0.05 and *p* = 0.04, respectively) (Figure 1B). A trend towards greater enzyme activities was appreciated in R group when compared with the C group (+47.9%; *p* = 0.078) (Figure 1B). In gastrocnemius muscle, no changes in the activity of this enzyme were found among the four experimental groups (Figure 2B).

### 3.3. Western Blot Analysis in IBAT and Skeletal Muscle

**Figure 3:**
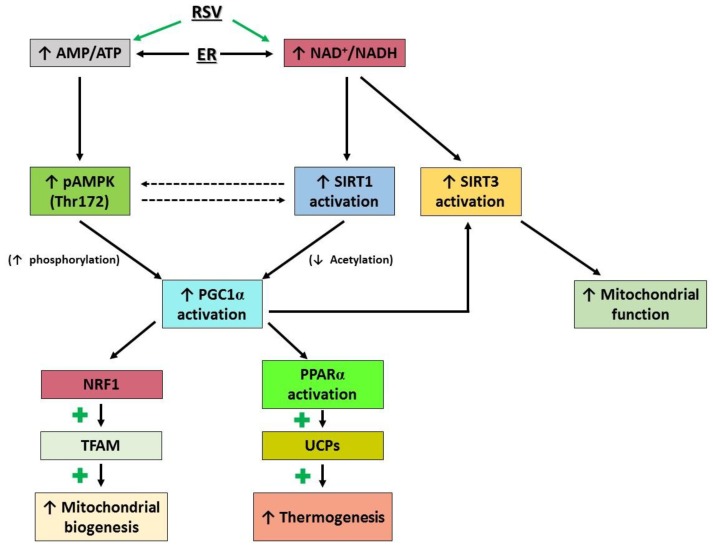
Effects of resveratrol and energy restriction in thermogenesis and mitochondrial synthesis pathways. RSV: resveratrol, ER: energy restriction, AMP: adenosine monophosphate, ATP: adenosine triphosphate, NAD^+^: oxidized nicotinamide adenine dinucleotide, NADH: reduced nicotinamide adenine dinucleotide, AMPK: AMP activated protein kinase, SIRT1: sirtuin 1, SIRT3: sirtuin 3, PGC1α: peroxisome proliferator-activated receptor gamma coactivator 1-alpha, NRF1: nuclear respiratory factor 1, TFAM: mitochondrial transcription factor A, PPAR α: peroxisome proliferator-activated receptor alpha, UCP: uncoupling protein.

In order to analyze the effects induced by resveratrol administration, energy restriction and the combination on IBAT and skeletal muscle thermogenic capacities and fatty acid oxidation, the expression or the activation of different proteins was analyzed (Figure 3).

**Figure 4:**
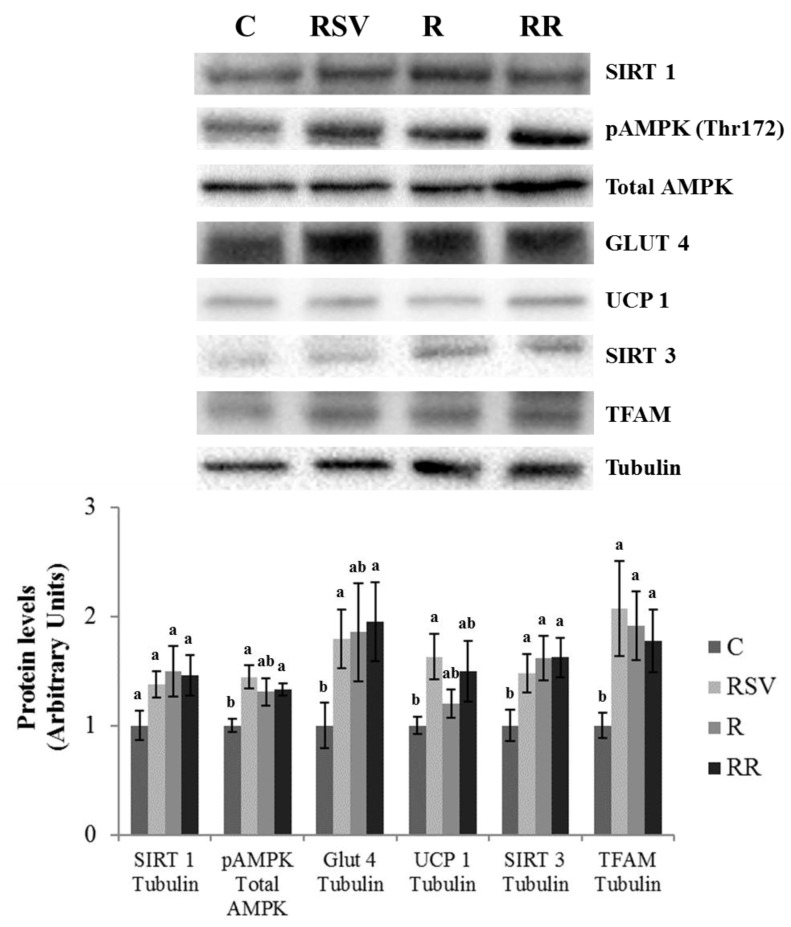
Protein levels of SIRT1, GLUT4, UCP1, SIRT3 and TFAM, and threonine 172 phosphorylation ratio of AMPK in IBAT of rats fed an obesogenic diet for 6 weeks and then shifted to a standard diet (C), or standard diet supplemented with resveratrol (RSV), or submitted to energy restriction and fed a standard diet (R), or submitted to energy restriction and fed a standard diet supplemented with resveratrol (RR) (*n* = 9/group) for additional 6 weeks. Values are represented as means ± SEM. Differences among groups were determined by using one-way ANOVA followed by Newman–Keuls post hoc test. Values not sharing a common letter are significantly different (*p* < 0.05). SIRT1: sirtuin 1, GLUT4: glucose transporter 4, UCP1: uncoupling protein 1, SIRT3: sirtuin 3, TFAM: mitochondrial transcription factor A, AMPK: AMP activated protein kinase, IBAT: interscapular brown adipose tissue.

**Figure 5:**
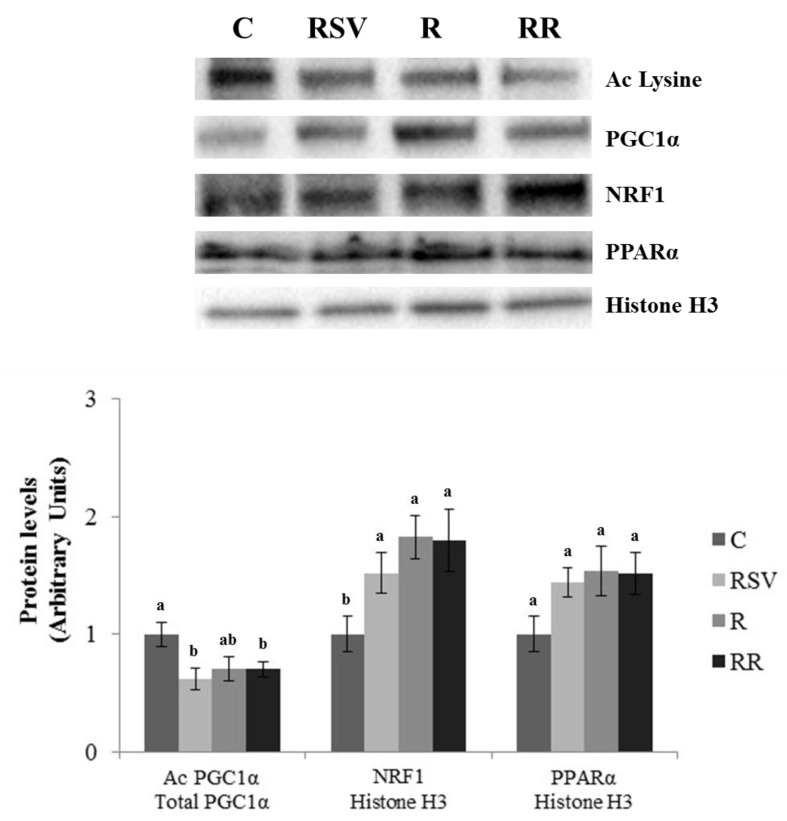
Acetylation ratio of PGC1α and protein levels of NRF1 and PPARα in IBAT of rats fed an obesogenic diet for 6 weeks and then shifted to a standard diet (C), or standard diet supplemented with resveratrol (RSV), or submitted to energy restriction and fed a standard diet (R), or submitted to energy restriction and fed a standard diet supplemented with resveratrol (RR) (*n* = 9/group) for additional 6 weeks. Values are represented as means ± SEM. Differences among groups were determined by using one-way ANOVA followed by Newman–Keuls post hoc test. Values not sharing a common letter are significantly different (*p* < 0.05). PGC1α: peroxisome proliferator-activated receptor gamma coactivator 1-alpha, NRF1: nuclear respiratory factor 1, PPARα: peroxisome proliferator-activated receptor alpha, IBAT: interscapular brown adipose tissue.

In IBAT, increased UCP1 protein expression was found in the group supplemented with resveratrol (RSV) in comparison with the C group (*p* = 0.03), as well as a tendency towards higher values in the RR group (+49.9%; *p* = 0.10) and no changes in the R group (Figure 4). In order to determine the molecular mechanisms underlying this effect, PGC1α, PPARα and SIRTs were studied. Decreased PGC1α acetylation, and thus greater activation of this protein, was found in the RSV and RR groups when compared with the C group (*p* = 0.02 and *p* = 0.03, respectively). In the case of R group, a tendency towards lower acetylation (−29.5%; *p* = 0.06) was observed (Figure 5). Regarding PPARα, although no differences in protein expression were found in the C group and the three treated groups, trends towards increased expressions were appreciated (+43.6%; *p* = 0.055 in the RSV group, +59.3%; *p* = 0.073 in the R group and +51.8%; *p* = 0.053 in the RR group) (Figure 5). Similarly, no significant changes were observed among the four groups in SIRT1 protein expression, but trends towards increased protein levels were found in the three treated groups when compared with the C group (+37.6%; *p* = 0.057 in the RSV group, +49.6%; *p* = 0.094 in the R group and +45.7%; *p* = 0.065 in the RR group) (Figure 4). As far as SIRT3 is concerned, greater protein levels were found in the three treated groups when compared with the C group (*p* = 0.05 in RSV, *p* = 0.033 in R and *p* = 0.019 in RR), with no differences among them (Figure 4). Finally, the phosphorylation ratio of threonine 172 residue was measured in AMPK to analyze its activation status. The groups supplemented with resveratrol (RSV and RR) showed greater phosphorylation ratios in comparison with the C group (*p* = 0.022 and *p* = 0.045, respectively), without differences between them. In the case of the R group, no significant change in the phosphorylation ratio (and thus, in the activity) was found (Figure 4).

Mitochondriogenesis is a process closely related to thermogenesis. Thus, proteins involved in this process were also analyzed. Increased protein expressions of NRF1 were found in the RSV, R and RR groups when compared with the C group (*p* = 0.04, *p* = 0.01 and *p* = 0.02, respectively), with no differences among them (Figure 5). A similar pattern of response was observed in TFAM protein levels (*p* = 0.04 in the RSV group, *p* = 0.02 in the R group and *p* = 0.04 in the RR group) (Figure 4). In addition, GLUT4 protein level, a glucose transporter that provides with this energy substrate to IBAT, was increased only in the groups supplemented with resveratrol (RSV and RR) (*p* = 0.048 and *p* = 0.050, respectively) (Figure 4). 

**Figure 6:**
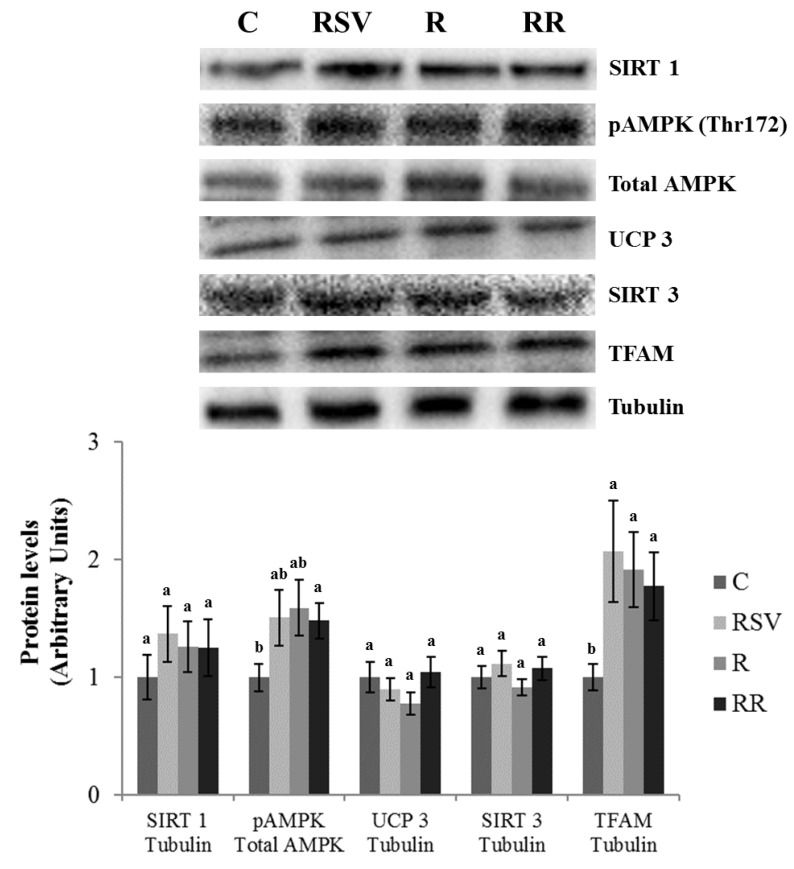
Protein levels of SIRT1, UCP3, SIRT3 and TFAM, and threonine 172 phosphorylation ratio of AMPK in gastrocnemius muscle of rats fed an obesogenic diet for 6 weeks and then shifted to a standard diet (C), or standard diet supplemented with resveratrol (RSV), or submitted to energy restriction and fed a standard diet (R), or submitted to energy restriction and fed a standard diet supplemented with resveratrol (RR) (*n* = 9/group) for additional 6 weeks. Values are represented as means ± SEM. Differences among groups were determined by using one-way ANOVA followed by Newman–Keuls post hoc test. Values not sharing a common letter are significantly different (*p* < 0.05). SIRT1: sirtuin 1, UCP3: uncoupling protein 3, SIRT3: sirtuin 3, TFAM: mitochondrial transcription factor 1, AMPK: AMP activated protein kinase.

**Figure 7:**
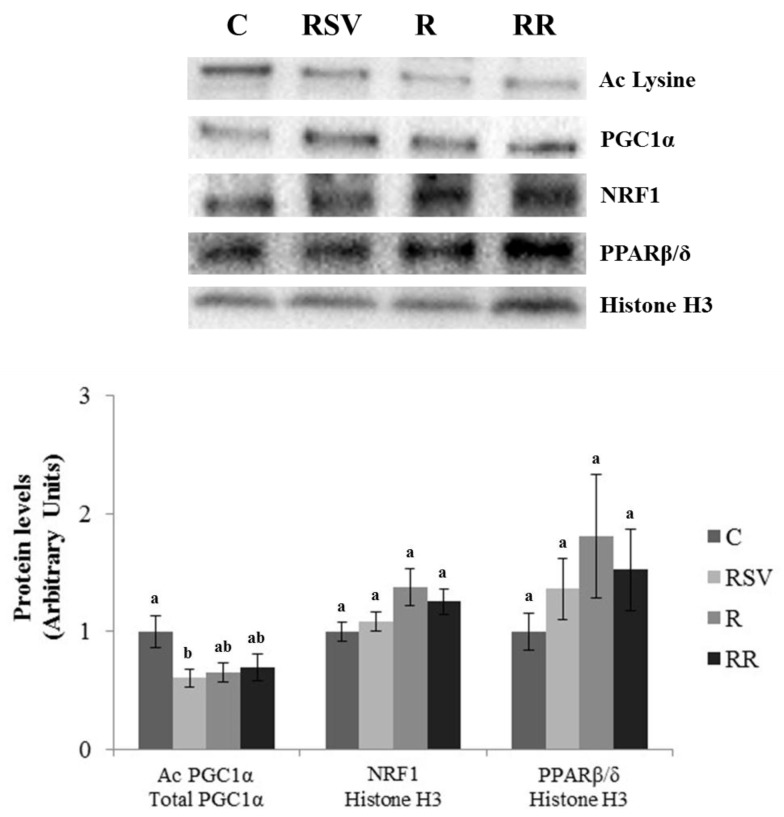
Acetylation ratio of PGC1α and protein levels of NRF1 and PPARβ/δ in gastrocnemius muscle of rats fed an obesogenic diet for 6 weeks and then shifted to a standard diet (C), or standard diet supplemented with resveratrol (RSV), or submitted to energy restriction and fed a standard diet (R), or submitted to energy restriction and fed a standard diet supplemented with resveratrol (RR) (*n* = 9/group) for additional 6 weeks. Values are represented as means ± SEM. Differences among groups were determined by using one-way ANOVA followed by Newman–Keuls post hoc test. Values not sharing a common letter are significantly different (*p* < 0.05). PGC1α: peroxisome proliferator-activated receptor gamma coactivator 1-alpha, NRF1: nuclear respiratory factor 1, PPAR β/δ: peroxisome proliferator-activated receptor beta/delta.

In the case of gastrocnemius muscle, no effects of the treatments were found in UCP3 protein expression (Figure 6). Regarding PGC1α, significantly lower acetylation, and thus greater activation of the protein, was observed in the RSV group in comparison to the C group (*p* = 0.03). In the case of restricted groups, only trends towards lower acetylation status were appreciated (−34.7%; *p* = 0.053 in R and −30.4%; *p* = 0.10 in RR) (Figure 7). Protein expressions of PPARβ/δ, SIRT1 and SIRT3 remained unchanged (Figure 6 and Figure 7). Similarly, lack of change was also found in NRF1, although a trend towards increased protein expressions was appreciated in the groups submitted to energy restriction when compared to the C group (+34.7%; *p* = 0.065 in the R group and +25.5%; *p* = 0.081 in the RR group) (Figure 7). By contrast, greater protein expression of TFAM was observed in the three treated groups in comparison with the C group (*p* = 0.04 in RSV group, *p* = 0.02 in the R group and *p* = 0.04 in the RR group) (Figure 6). Finally, significantly greater activation of AMPK was only observed in the RR group when compared with the C group (*p* = 0.024). In the case of the RSV and R groups, tendencies towards an increased activation were found (+50.9%; *p* = 0.082 and +58.9%; *p* = 0.054, respectively) (Figure 6). 

## 4. Discussion

As indicated in the introduction, the interest of the scientific community in natural bioactive compounds with beneficial health effects has been increasing in recent years. Regarding resveratrol, positive effects on several prevalent diseases have been reported both in animals and humans [19,31,32,33,34]. It is important to emphasize that, with regard to its effects on obesity and related co-morbilities such as insulin resistance and liver steatosis, the vast majority of the reported studies addressed in animal models have been carried out by using experimental designs in which resveratrol was administered together with an obesogenic diet. Consequently, the beneficial effects observed in these studies were related to the prevention of weight gain and metabolic alterations induced by this dietary pattern. However, the applicability of such experimental design in humans is unlikely. Indeed, for ethical reasons, it is not possible to recommend people to take resveratrol while they maintain an inappropriate dietary pattern.

Bearing this in mind, we focused our interest on the potential beneficial effects of resveratrol as a tool for obesity treatment. Thus, we assessed its effects on rats that had previously developed overweight/obesity (induced by diet). For this purpose, two approaches were designed. In the first, once obesity induction had stopped resveratrol was administered, as the single tool to treat obesity, together with a standard diet, while in the other resveratrol was combined with an energetically restricted diet, which is the most commonly strategy used in obesity treatment

It is important to point out that, in the vast majority of the reported studies, energy restriction ranges from 20% to 40%. In the present study a lower degree of restriction (15%) was chosen. The reason was based on a previous study from our group [35]. In that study we looked for synergistic effects between resveratrol, at a dose of 30 mg/kg of body weight/day, and 25% energy restriction. We observed that the addition of resveratrol to the restricted diet did not lead to additional reductions in fat mass or in serum insulin concentrations with regard to those produced by energy restriction alone. We believed that one of the reasons that could explain this situation was that the effects caused by energy restriction were strong enough to mask the potential positive effects ascribed to resveratrol. Consequently, a lower degree of energy restriction was preferred in the present study.

In IBAT, resveratrol administration increased UCP1 protein expression, suggesting a greater thermogenic capacity. In order to analyze the mechanism underlying this effect, we studied the regulation pathway described in Figure 3. Resveratrol induced deacetylation, and thus activation, of PGC1α, a co-activator of PPARα, which in turn regulates the expression of UCP1. It has been reported that SIRT1 is the main enzyme responsible for PGC1α deacetylation [36]. In the present study, a tendency towards increased protein expression of SIRT1 was observed. Although its activation was not directly measured, the deacetylation of PGC1α suggests that it was in fact activated. In addition, resveratrol increased the activity of AMPK, an enzyme that phosphorylates PGC1α, thus enhancing its activity. Furthermore, SIRT3 is activated by PGC1α, mediating the effects of this sirtuin on mitochondrial function and synthesis [37,38]. Moreover, the expression of this deacetylase has been reported to be positively correlated with a variety of mitochondrial proteins, UCP1 among them [39]. In the case of our study, significantly increased protein expression of SIRT3 was observed in resveratrol-treated rats. These results show that resveratrol increased the expression of UCP1 by activating the SIRT1/PGC1α/PPARα axis.

Fatty acid oxidation is an important part of the thermogenic program. It has been reported that the activation of PPARα by PGC1α leads to increased NRF1 and the subsequent increase in the synthesis of TFAM, which in turn results in enhanced duplication of mitochondrial DNA (Figure 3). Accordingly, in the present study PGC-1α activation was indeed accompanied by increases in NRF1 and TFAM expressions, suggesting that resveratrol, under the present experimental conditions, enhanced mitochondriogenesis. This is in good accordance with the increased activities of CPT1a and CS observed in rats treated with resveratrol. BAT can also use glucose as an energetic substrate. In the present study resveratrol induced a significant increase in GLUT4 protein expression, meaning that the capacity of IBAT to uptake glucose was greater in rats treated with this phenolic compound. Taken as a whole, these results show that resveratrol did not only increase the key factor for thermogenesis (UCP1) but also the availability of substrates to keep this process activated. 

In previous studies from our laboratory we had already observed the induction of UCP1 by resveratrol in IBAT [22,40] and other authors had also reported similar results [41,42]. In all these studies the animals received the phenolic compound at the same time as an obesogenic diet, during the fattening period. By contrast, in this work the animals were fed a high-fat high sucrose diet to become obese and then they were shifted to a standard diet, and treated with resveratrol. Taken together, the present results and those reported previously demonstrate the ability of resveratrol to induce thermogenesis under both overfeeding and normal feeding conditions. This may represent a mechanism of action underlying the beneficial effect of resveratrol in obesity management. 

As previously mentioned, a second approach in this study was to analyze the effects induced by resveratrol under energy restriction. In fact, the usefulness of this phenolic compound in combination with the most common strategy for obesity treatment was intended to analyze whether potential additive or synergistic effects could take place. This is an interesting approach because it would result in the same positive effect obtained by following a more restricted diet non supplemented with resveratrol, whose compliance is more difficult to achieve. 

For this purpose, first of all we analyzed the effects of energy restriction on the parameters previously studied in the first approach. No change in UCP1 protein expression was observed in R group. By contrast, NRF1 and TFAM protein expressions, as well as the CPT1a activity were significantly increased in these animals, suggesting that mitochondrial synthesis and function were enhanced. The increased SIRT3 protein expression observed in this experimental group supports the idea of an ameliorated mitochondrial function, in good accordance with previous data describing the activation of this protein in different tissues under energy restriction conditions [43,44,45]. It is important to remember that resveratrol has been suggested as an energy restriction mimetic [20,21,46,47]. However, as shown by the present results, differential effects on thermogenic capacity are found between them.

When we analyzed the RR group, in general terms, the effects on the parameters studied were similar to those observed in the RSV group, although in this case only a trend towards higher values was observed in UCP1. These results demonstrate that the administration of resveratrol in combination with energy restriction does not provide any advantage.

In the case of skeletal muscle, the effects of resveratrol were not as clear as in IBAT. The administration of this polyphenol was not able to increase protein expression of UCP3, despite the activation of PGC-1α. Nevertheless, increased TFAM protein expression and CPT-1b activity suggest increased fatty acid oxidation, associated to enhanced mitochondrial synthesis. It has been reported that the activation of PGC-1α is based on sirtuin-mediated deacetylation and phosphorylation by AMPK [48,49]. In the case of skeletal muscle, the absence of any significant activation of AMPK by resveratrol may explain the lack of activation of every single step in mitochondriogenesis pathway, and consequently in CS activity. 

In the studies reported by other authors there is no consensus regarding the effects of resveratrol on skeletal muscle mitochondrial biogenesis induction. Indeed, while an enhanced mitochondrial synthesis and/or function has been described in studies where the compound was administered in conditions of certain metabolic stress [41,50,51], this effect seems to disappear when the polyphenol is administered in healthy subjects [52,53,54]. Regarding UCP3, the lack of change found in this study is not in good accordance with data reported by other authors. In those studies, resveratrol effectively induced UCP3 mRNA or protein expression in the skeletal muscle of mice and rats fed in obesogenic diets [22,41]. Moreover, greater UCP3 protein expression was also described in this tissue in mice fed in a standard diet and supplemented with the polyphenol [55]. Nevertheless, the discrepancy between our results and those previously reported could result from significant differences in the experimental design (animal model, experimental period length, resveratrol dose and diet composition). 

As far as energy restriction is concerned, no relevant significant changes were observed in the parameters analysed. By revising data reported in the literature concerning this issue we observed that some controversy exists. Thus, while some authors have reported that the maintenance of a 30% energy restriction for a period of 3 months effectively induces mitochondrial biogenesis and function (increased respiration and expression of genes important for oxidative function), others have found no such effects under similar experimental conditions [56,57]. In fact, in a study conducted in mice of different ages (6, 12 and 24 month old B6D2F1 mice) that underwent a lifelong energy restriction (40%), the authors concluded that energy restriction did not induce mitochondrial protein synthesis, but maintained this parameter while decreasing cellular proliferation [58]. Moreover, the authors point towards the techniques used by Nisoli et al., and Hancock et al. in determining the effects of energy restriction on mitochondrial synthesis (mRNA and protein expression of mitochondrial proteins) as being the cause of their contradictory results. Finally, when resveratrol was administered under energy restriction feeding conditions, the results were very similar to those observed when the compound was administered under standard feeding conditions. 

We would like to point out that in previous studies conducted in this precise cohort of rats aimed at studying the effects of the aforementioned treatments on glucose homeostasis and hepatic steatosis ([29,30], respectively), resveratrol-induced effects were less marked than those produced by energy restriction. By contrast, when considering the effects on UCP expression and fatty acid oxidative capacity, as in the present study, resveratrol was more effective than a mild energy restriction. 

In conclusion, in obese rats under standard feeding conditions, resveratrol is able to increase thermogenic and oxidative capacities in IBAT. This feature represents a mechanism by which this phenolic compound could show an anti-obesity action. Nevertheless, more research is needed in order to interpret the importance of this mechanism. In the case of skeletal muscle, oxidative capacity is increased by this polyphenol intake. The administration of resveratrol in combination with energy restriction apparently does not provide any advantage.

## Figures and Tables

**Table 1:** Weights of IBAT, gastrocnemius muscle and subcutaneous, perirenal, mesenteric and epididymal adipose tissues of rats fed on the experimental diets for 6 weeks.

	C	RSV	R	RR	ANOVA
IBAT (g)	0.76 ± 0.07	0.83 ± 0.03	0.83 ± 0.03	0.82 ± 0.06	NS
Gastrocnemius (g)	2.42 ± 0.03	2.65 ± 0.16	2.62 ± 0.10	2.60 ± 0.08	NS
Subcutaneous AT (g)	14.6 ± 1.1ab	15.2 ± 0.9a	12.0 ± 0.6bc	10.4 ± 0.6c	(*p* < 0.01)
Perirenal AT (g)	14.6 ± 1.4a	13.6 ± 0.8a	8.7 ± 0.3b	9.3 ± 0.5b	(*p* < 0.05)
Mesenteric AT (g)	3.9 ± 0.4ab	4.3 ± 0.1a	2.9 ± 0.3bc	268 ± 0.2c	(*p* < 0.05)
Epididymal AT (g)	11.0 ± 0.9a	12.4 ± 0.2a	7.4 ± 0.5b	7.1 ± 0.5b	(*p* < 0.01)

IBAT, interscapular brown adipose tissue; AT, adipose tissue. Values are mean ± standard error of the mean (SEM). Differences among groups were determined by using one-way analysis of variance (ANOVA) followed by Newman Keuls post-hoc test. Values not sharing a common letter are significantly different. NS: Not significant.

## Supplementary Materials

The following are available online at http://www.mdpi.com/2072-6643/10/10/1446/s1, Figure S1: Timeline of the complete experimental period (12 weeks), Table S1: Composition of the experimental (A) High-fat High-sucrose and (B) Standard control diets, Table S2: References of the antibodies used for western blot analyses.
