# Supplementary material for: Do the Effects of Resveratrol on Thermogenic and Oxidative Capacities in IBAT and Skeletal Muscle Depend on Feeding Conditions?
Source: Nutrients. 2018 Oct 6;10(10):1446. doi: 10.3390/nu10101446 (PMC6213586; doi:10.3390/nu10101446)
Supplement: Supplementary file 1 [file nutrients-10-01446-s001.pdf]

SUPPLEMENTAL MATERIAL. Do the effects of resveratrol on thermogenic and oxidative capacities in IBAT and skeletal muscle depend on feeding conditions?

I. Milton-Laskibar[1,5], L. Aguirre[1,5*], U. Etxeberria[2,3], F.I. Milagro[4,5], J.A. Martínez[4,5,6] and M.P. Portillo[1,5]

[1]*Nutrition and Obesity Group. Department of Nutrition and Food Science, University of the Basque Country (UPV/EHU) and Lucio Lascaray Research Institute, Vitoria, Spain*

[2]*BCC Innovation. Technological Center of Gastronomy, Donostia-San Sebastián, Spain*

[3]*Basque Culinary Center. Mondragon Unibertsitatea, Donostia-San Sebastian, Spain*

[4]*Department of Nutrition, Food Sciences and Physiology, Centre for Nutrition Research, University of Navarra, Pamplona, Spain*

[5]*CIBERobn Physiopathology of Obesity and Nutrition, Institute of Health Carlos III, Spain*

[6]*IMDEA Food, Madrid, Spain*

***Corresponding author:** Leixuri Aguirre. Dpt. Nutrition and Food Science. Faculty of Pharmacy. University of the Basque Country. Paseo de la Universidad, 7. 01006 Vitoria Gasteiz (Spain). Phone: +34-013863. Fax: +34-945013014.

e-mail: leixuri.aguirre@ehu.eus.

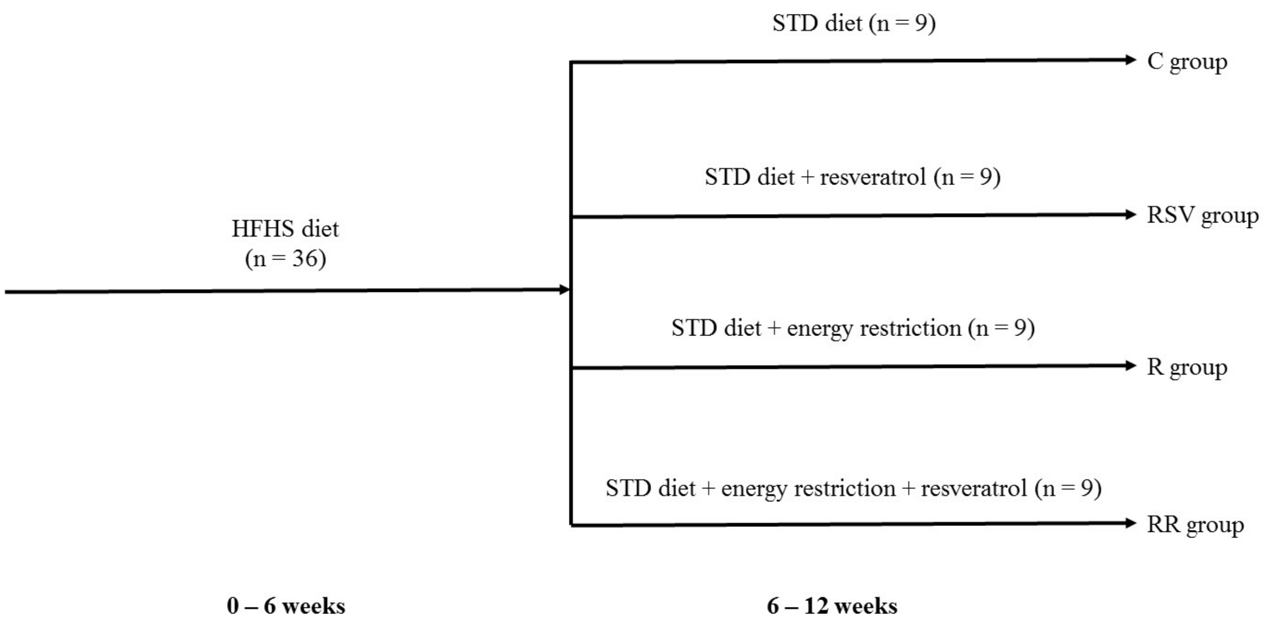

Figure S1. Timeline of the complete experimental period (12 weeks). HFHS = High-fat high-sucrose diet; STD = standard diet.

Table S1. Composition of the experimental (A) High-fat High-sucrose and (B) Standard control diets.

A (OpenSource Diets, Lynge, Denmark; Ref. D12451)

**Ingredient**	**Weight (g)**	**Energy (kcal)**
Casein, 80 Mesh	200	800
L-Cystine	3	12
Corn Starch	72.8	291
Maltodextrin 10	100	400
Sucrose	172.8	691
Cellulose, BW200	50	0
Soybean Oil	25	225
Lard	177.5	1598
Mineral Mix S10026	10	0
DiCalcium Phosphate	13	0
Calcium Carbonate	5.5	0
Potassium Citrate, 1 H2O	16.5	0
Vitamin Mix V10001	10	40
Choline Bitartrate	2	0
FD&C Red Dye #40	0.05	0
Total	858.15	4057

B (OpenSource Diets, Lynge, Denmark; Ref. D10012G)

**Ingredient**	**Weight (g)**	**Energy (kcal)**
Casein, 30 Mesh	200	800
L-Cystine	3	12
Corn Starch	397	1590
Maltodextrin	132	528
Sucrose	100	400
Cellulose	50	0
Soybean Oil	70	630
t-Butylhydroquinone	0.014	0
Mineral Mix S10022G	35	0
Vitamin Mix V10037	10	40
Choline Bitartrate	2.5	0
Total	1000	4000

Table S2. References of the antibodies used for western blot analyses.

Protein	Manufacturer	Catalogue reference
SIRT1	Abcam	ab110304
SIRT3	Santa Cruz	SC-365175
AMPK (Thr172)	Cell Signaling	#2535
AMPK	Cell Signaling	#2532
UCP1	Santa Cruz	SC-6529
UCP3	Santa Cruz	SC-7756
GLUT4	Santa Cruz	SC-1608
TFAM	Santa Cruz	SC-23588
NRF1	Abcam	ab175932
α Tubulin	Cell Signaling	#2125
PGC1 α	Abcam	ab54481
PPAR α	Abcam	ab24509
PPAR β/δ	Santa Cruz	SC- 7197
Histone H3	Cell Signaling	#9715
Mouse (IgG-HRP)	Santa Cruz	SC-2031
Rabbit (IgG-HRP)	Santa Cruz	SC-2004
